# A robust method for automatic identification of landmarks on surface models of the pelvis

**DOI:** 10.1038/s41598-019-49573-4

**Published:** 2019-09-16

**Authors:** Maximilian C. M. Fischer, Felix Krooß, Juliana Habor, Klaus Radermacher

**Affiliations:** Chair of Medical Engineering, Helmholtz-Institute for Biomedical Engineering, RWTH Aachen University, Aachen, Germany

**Keywords:** Bone, Computational biophysics, Software

## Abstract

The recognition of bony landmarks of the pelvis is a required operation in patient-specific orthopedics, subject-specific biomechanics or morphometrics. A fully automatic detection is preferable to a subjective and time-consuming manual identification. In this paper, a new approach, called the iterative tangential plane (ITP) method, for fully automatic identification of landmarks on surface models of the pelvis is introduced. The method includes the landmarks to construct the two most established anatomical reference frames of the pelvis: the anterior pelvic plane (APP) coordinate system and superior inferior spine plane (SISP) coordinate system. The ITP method proved to be robust against the initial alignment of the pelvis in space. A comparison to a manual identification was performed that showed minor but significant (p < 0.05) median differences below 3 mm for the position of the landmarks and below 1° for the orientation of the APP coordinate system. Whether these differences are acceptable, has to be evaluated for each specific use case. There were no significant differences for the orientation of the SISP coordinate system recommended by the International Society of Biomechanics.

## Introduction

The identification of bony landmarks is an essential procedure in patient-specific orthopedics, subject-specific biomechanics or morphometrics. Fields of application are for instance patient-specific preoperative planning^1^ and intraoperative navigation^2,3^, subject-specific scaling of musculoskeletal models^4,5^ or the statistical analysis of the bony morphology^6–9^. The landmarks are used to construct subject-specific anatomical reference frames^10,11^ and to quantitatively determine the morphology of bones. Manual identification of landmarks is time-consuming^1,12^, requires medical training, is subject to intra- and inter-observer variability^13,14^ and is not suitable for the analysis of large data sets with many subjects. Automatic methods for landmark identification should provide reproducible results, be invariant against the orientation and position of the bone and robust against the large inter-subject variability of the bones. While the hip joint, with the acetabulum being part of the pelvis, is one of the most investigated joints of the human body, only a few methods for fully automatic identification of pelvic landmarks on surface models have been published so far.

## Related Work

A list of all abbreviations used in this paper can be found as Supplementary Table S1.

Ehrhardt *et al*. proposed an atlas-based approach with a curvature-based refinement of the landmarks^1^. The atlas consists of gray value data and a surface model of the pelvis with labeled anatomical areas and landmarks. Initially, the atlas is non-rigidly registered to the computed tomography (CT) data of the subject using the gray value data. Afterwards, the atlas and the subject mesh are locally cut out within a certain radius for each landmark and the registration of each cut-out is refined by a combination of an affine and a non-linear registration algorithm taking the Euclidean distance, the normals and the curvature of the surfaces into account. However, a manually labeled atlas and CT data of the subject are necessary for this approach. The gray value-based initialization of the atlas is error-prone due to large anatomical variations between subjects. Moreover, the limited number of seven subjects and the missing comparison to a manual landmark identification impede the assessment of Ehrhardt’s method.

Seim *et al*. compared three methods for the identification of the landmarks of the anterior pelvic plane (APP). The APP is usually constructed by the anterior superior iliac spines (ASISs) and the pubic tubercles (PTs) or the pubic symphysis (PS). Seim *et al*. evaluated one convex hull-based method and two statistical shape model (SSM)-based methods^15^. All methods are based on a previous SSM-based segmentation and a graph-based optimized reconstruction of the subject’s pelvis from CT data. During this segmentation process, the pelvis is subdivided into regions such as the iliac and pubic bones. For the first method, the face of the convex hull of the pelvis with the vertices with minimal distance to the iliac and pubic regions defines the APP. This method is limited to landmarks that are part of the convex hull. The SSM-based methods only differ in the number of manually labeled data sets in the training data of the SSM. Both methods transfer the landmarks from the SSM to the optimized reconstruction of the subject’s pelvis because both meshes share the same topology. However, for all three methods of Seim *et al*. a sufficient amount of training data for the creation of the SSM and CT data of the subject is necessary. In their study, they used 50 datasets for the generation of the SSM.

In addition to Ehrhardt’s and Seim’s methods, further methods for the detection of pelvic landmarks on raw image data have been proposed^12,16,17^. In contrast, our study focuses on fully automatic pelvic landmark identification on surface models of the pelvis considering scenarios with many subjects from different sources, where the volume data might not be available for instance due to reasons of data protection. The following studies addressed this issue.

Subburaj *et al*. presented a curvature-based approach in combination with a spatial relationship matrix of the landmarks^18^. The surface of the mesh is grouped into different regions (peaks, ridges, pits and ravines) based on the curvature value. Subsequently, the regions were iteratively selected and labeled considering the spatial relationship matrix of the landmarks. However, the spatial relationship matrix depends on the alignment of the pelvis in space and the initialization of the spatial relationship matrix is unclear. Moreover, the approach was tested only for one subject. Our study will show that there is a trade-off between the detection rate and the accuracy depending on the number of landmarks in the spatial relationship matrix.

Several studies focused on the detection of the landmarks that are necessary to construct the APP coordinate system. Kai *et al*. proposed a method to identify the APP purely based on the surface of the pelvis^10^. The surface is transformed to its principal axes of inertia and subsequently subdivided into four parts by a sagittal and a transverse plane. The most anterior points of the four parts define the landmarks of the APP. However, due to the variability of the pelvic morphology, the principal axes do not ensure a unified orientation for all subjects. Hence, the most anterior points are not necessarily the landmark points of the APP, defined by placing the anterior side of the pelvis on a table. Higgens *et al*., Zhang *et al*. and Chen *et al*. presented an iterative refinement of the APP identification^6,19,20^. Still, none of the three approaches is fully automatic. They are based on an initial manual selection of the approximated landmarks of the APP.

In this paper, we introduce a fully automatic approach, hereafter referred to as iterative tangential plane (ITP) method, for identification of landmarks on a surface model of the pelvis. Additionally to the APP, the ITP method identifies the posterior superior iliac spines (PSISs), ischial spines (ISs) and the sacral promontory (SP). The ASISs and the midpoint between the PSISs define the superior iliac spine plane (SISP) recommended by the International Society of Biomechanics^11^.

Any automatic landmark identification has to take into account that the anatomical planes of the patient can highly deviate from the CT coordinate system and that the reference systems of medical imaging systems are not standardized. We hypothesized, that the ITP method robustly identifies the pelvic landmarks independently from the initial orientation or position of the surface model of the pelvis and without significant differences to a manual identification of the landmarks.

## Material and Methods

### Subject data

The CT data of twenty cadaveric subjects from the open source virtual skeleton database^21^ hosted at the SICAS Medical Image Repository (www.smir.ch) were used in this study (Table 1). Subjects with obvious bone fractures of the pelvis or metal artifacts in the region of the pelvis were not included in the study. Additional information for each subject can be found in the Supplementary Table S2.

**Table 1:** Gender and age of the cadaveric subjects.

Gender	Number of subjects	Age		
		Median	IQR (Q1 to Q3)	Range (min. to max.)
Male	13	58	35 (38 to 73)	59 (25 to 84)
Female	7	41	14 (38 to 52)	31 (30 to 61)

The surface of each pelvis was semi-automatically reconstructed by thresholding followed by a manual post-processing using the software 3D Slicer (www.slicer.org) with the default smoothing settings. If necessary, the pelvic bones were manually segmented at the PS and the sacroiliac joints. The reconstructed surfaces were imported into MATLAB using a conservative decimation and remeshing procedure. The decimator restricted the Hausdorff distance between input and output mesh to 0.05 mm. The adaptive remesher permitted a maximum deviation of 0.05 mm from the input mesh with a minimum edge length of 0.5 mm and a maximal edge length of 100 mm. The decimator and remesher are plugins of the software OpenFlipper (www.openflipper.org).

### Pelvic landmark identification

#### ITP method

**Figure 1:**
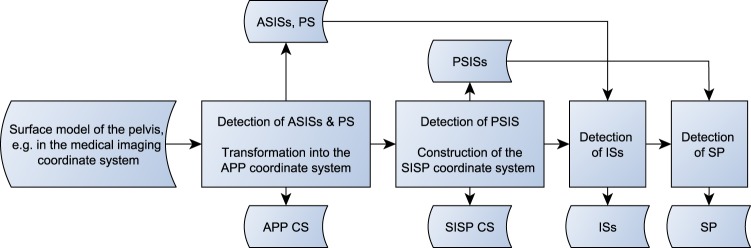
Flowchart of the ITP method.

A general overview of the ITP method is depicted as flowchart in Fig. 1.

The ITP method starts with a translation of the surface mesh to its center of mass^22^ and a rotation to its principal axes of inertia, the eigenvectors of the tensor of inertia sorted by the eigenvalues in descending order^10^. Due to the morphology of the pelvis, this leads in the majority of cases to the required orientation of the temporary pelvic coordinate system (TPCS) as listed in Table 2. However, the transformation to the principal axes of inertia does not guarantee the required orientation for every pelvis due to the large inter-individual variability of the pelvic morphology.

**Table 2:** Required orientation of the temporary pelvic coordinate system (TPCS) after the rotation of the pelvis to its principal axes of inertia.

Axis	x	y	z
Positive direction	Right	Inferior	Anterior
Negative direction	Left	Superior	Posterior

Thus, two additional sanity checks are introduced. The maximal pelvic width is defined as the distance between the most lateral points in x-direction. At first, the y-coordinates of the points defining the maximal pelvic width have to be negative. If the y-coordinate of these points is positive, the TPCS is rotated as follows: If the z-coordinate of the point of the pelvis with the minimal distance to the z-axis is negative, the TPCS is rotated by 180° around the z-axis otherwise it is rotated by 180° around the x-axis. Secondly, the x-coordinate of the most posterior point of the pelvis has to be smaller than a quarter of the maximal pelvic width otherwise the TPCS is rotated by 180° around the y-axis. This value was empirically determined. These two sanity checks guarantee the required orientation of the TPCS (Table 2) and they were not described by Kai *et al*.

**Figure 2:**
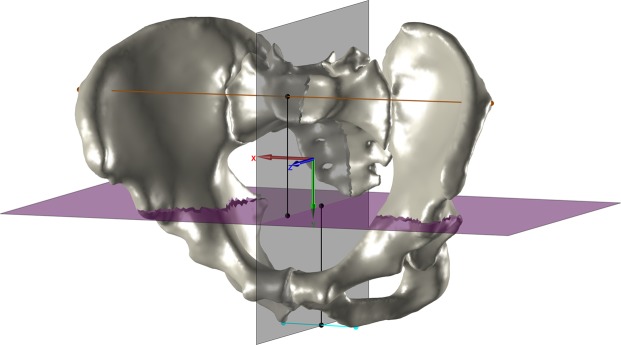
The pelvis of subject z001 in the temporary coordinate system. The maximal pelvic width is depicted in brown, the distal pelvic width in cyan. The sagittal plane (gray) and the transverse plane (magenta) section the pelvis into four quadrants. The distance of the transverse plane to the maximal and the distal pelvic width in the y-direction is illustrated by the two black lines that have the same length.

Subsequently, the sagittal plane and a transverse plane section the pelvis into four quadrants (Fig. 2). The position of the transverse plane is defined by the midpoint between the height of the maximal pelvic width and the distal pelvic width. The distal pelvic width connects the most distal points of the left and the right side. The height of the maximal pelvic width and the distal pelvic width is the y-coordinate of the intersection point of each width with the sagittal plane^10^. After this step, a check is performed to ensure that the most medial parts of both pubic bones are not part of the same distal quadrant. This procedure is described in the source code published together with this paper.

Now the landmarks of the APP can be iteratively identified. The most anterior point of each quadrant defines the temporary ASISs and PTs. The PS is defined as the projection of the midpoint of the shortest line connecting the pubic bones onto the line connecting both PTs. In order to determine the shortest line between the pubic bones, the pairwise distance is computed between all points of the distal quadrants and the shortest distance is selected. Usually the midpoint of the connection of the PTs is used as PS^10^, but this can lead to a lateral offset of the PS due to asymmetric PTs. The temporary ASISs and PS define the new TPCS. All quadrants are rotated into the TPCS and the detection of the landmarks is repeated until the TPCS converges to the unit matrix. This iterative refinement is not described by Kai *et al*. As a result, the final ASISs and PS are identified.

**Figure 3:**
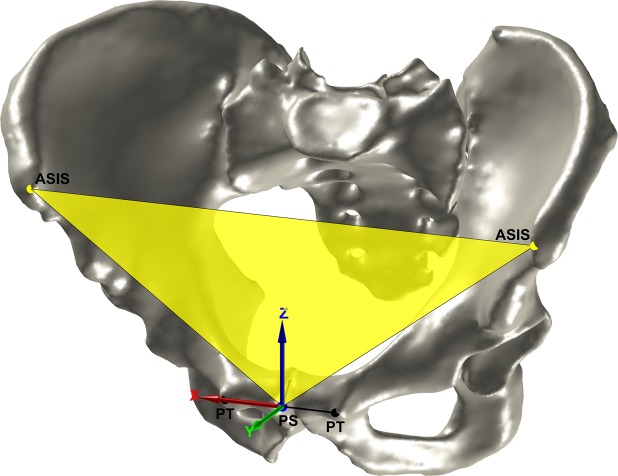
The pelvis in the APP coordinate system. The APP and ASISs are depicted in yellow. The PTs and the construction of the PS are depicted in black. The PS is depicted in blue.

For the detection of the other landmarks, the mesh is transformed into the APP coordinate system. The origin is the PS, the x-axis is defined by the vector connecting the ASISs, the y-axis is the normal of the APP, and the z-axis is orthogonal to the x-axis and the y-axis (Fig. 3).

The PSISs are identified in a similar way as the ASISs. The most posterior point of each hip bone defines the temporary PSISs. The ASISs and the midpoint between the temporary PSISs define the new TPCS. The pelvis is rotated into the TPCS and the detection of the landmarks is repeated until TPCS converges to the unit matrix. In case of a missing segmentation of the sacroiliac joints or large osteophytes or bridging at the posterior inferior iliac spines (PIISs) additional sanity checks are necessary that are described in the source code published with this study.

**Figure 4:**
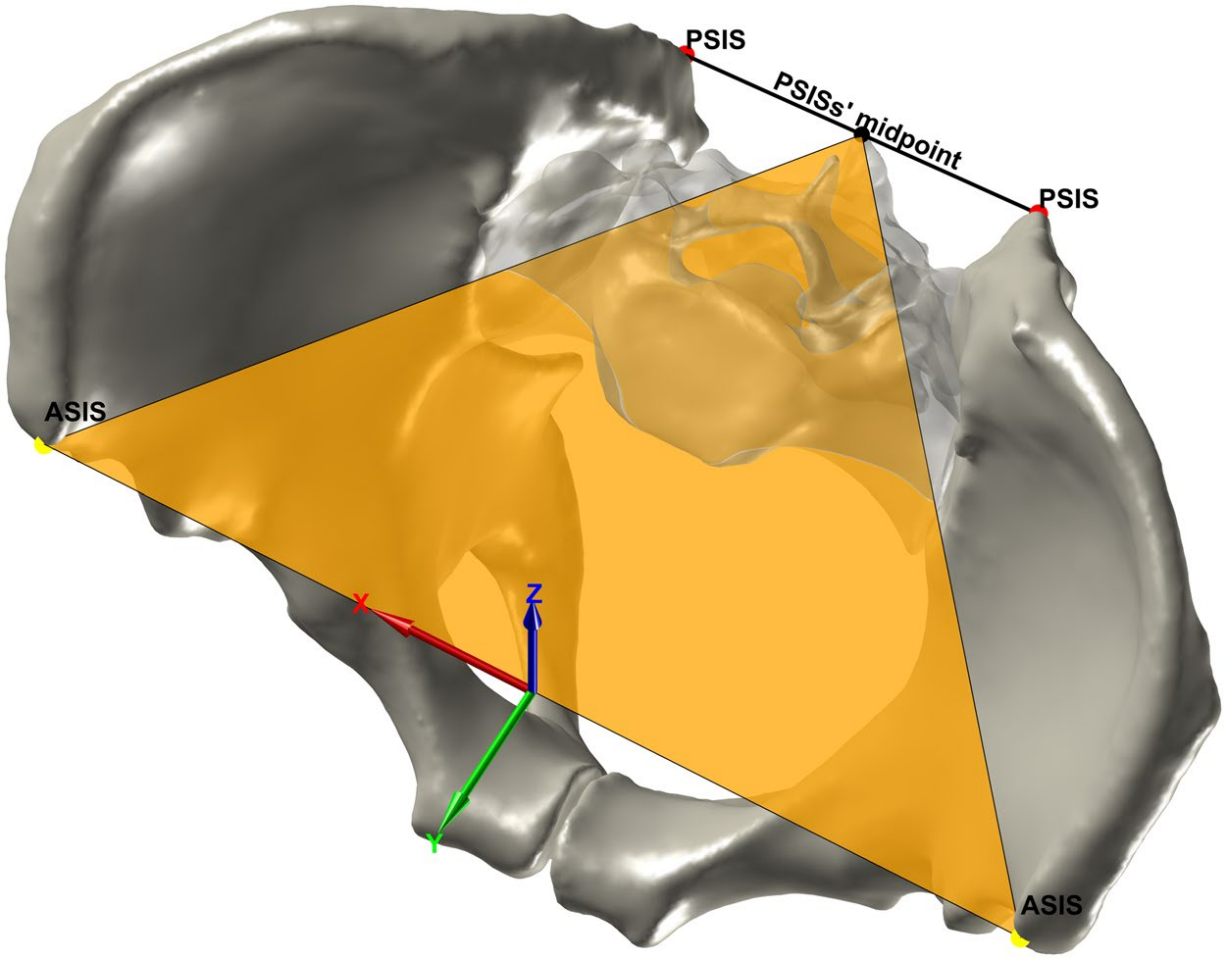
The identification of the PSISs and the SISP coordinate system. The PSISs are depicted in red, the PSISs’ midpoint in black and the SISP in orange.

The origin of the SISP coordinate system is the midpoint between the ASISs, the x-axis is defined by the vector connecting the ASISs, the z-axis is the normal of the SISP, and the y-axis is orthogonal to the x-axis and the z-axis (Fig. 4).

**Figure 5:**
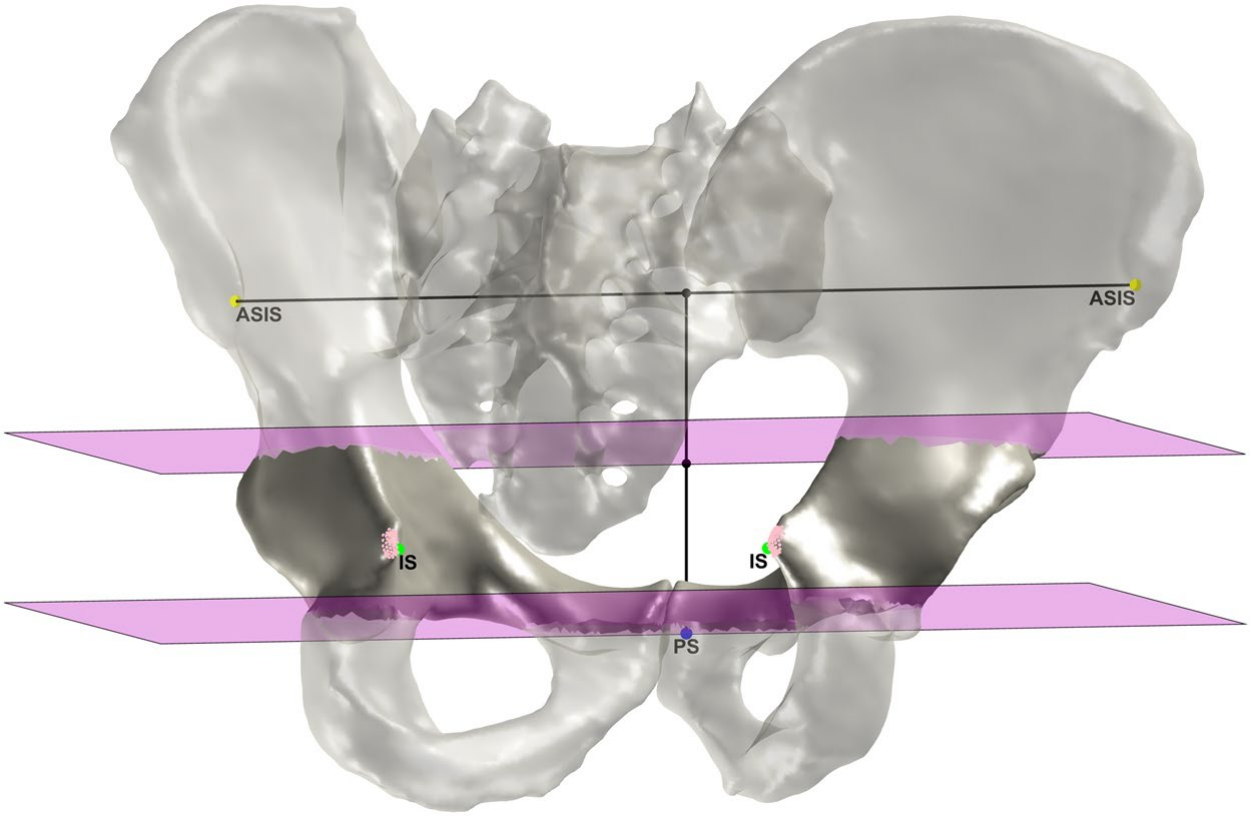
The identification of the ISs. The two transverse cutting planes are depicted in magenta, the most posterior points in pink and the final ISs in green.

The APP height is defined by the distance between PS and the projection of the PS on the line connecting the ASISs. The pelvis is cut along two transverse planes, a superior one at the middle of the APP height and an inferior one at the PS. The two parts of the hip bones between these planes contain the ISs. The parts are counter-clockwise rotated from −5° to 45° in 1° steps around the x-axis. For each step, the most posterior point of both parts is stored. The points of both parts with the minimal distance between each other define the ISs (Fig. 5).

**Figure 6:**
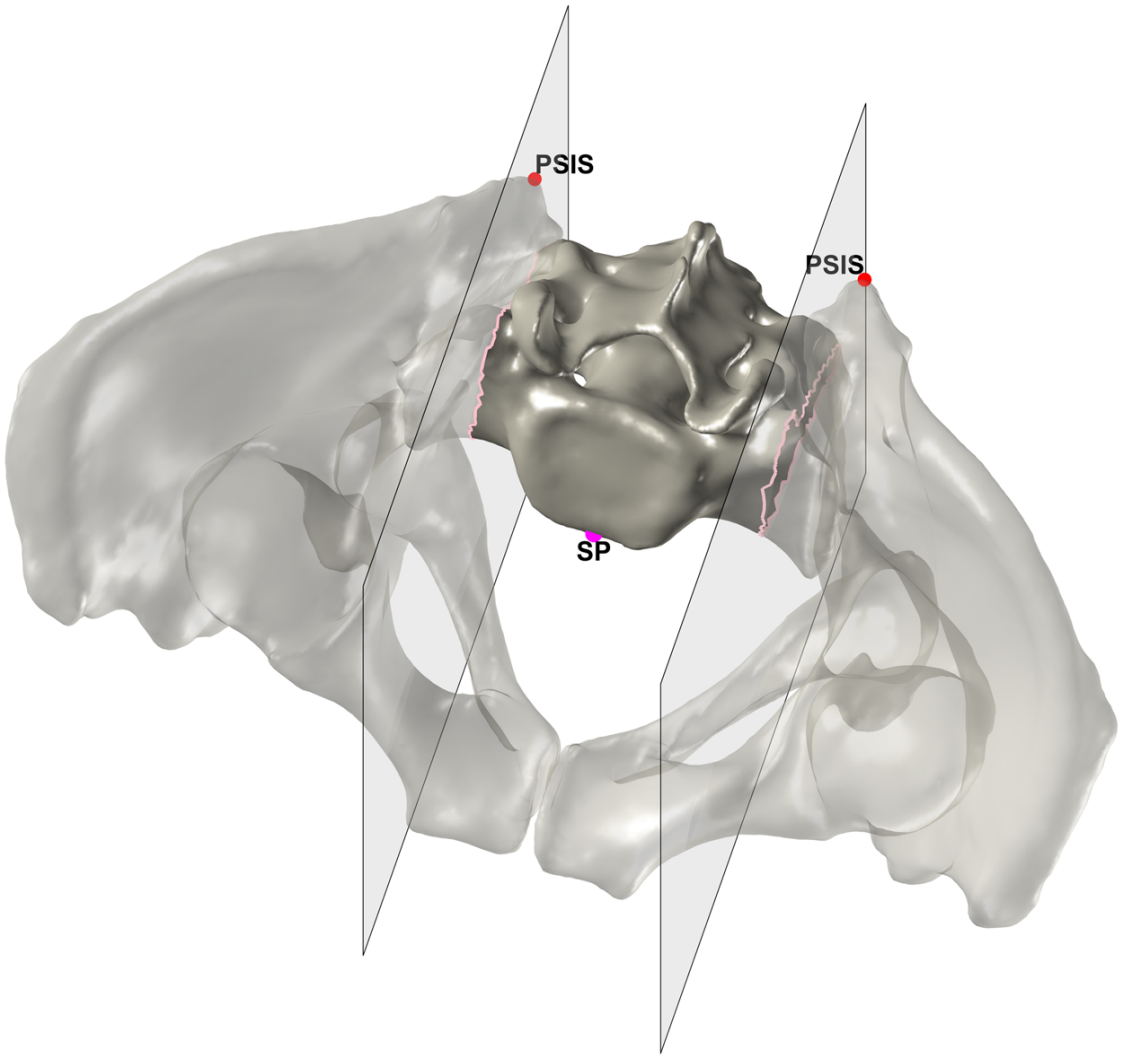
The identification of the SP. The PSISs are depicted in red, the two sagittal cutting planes in gray and the intersections of the cutting planes in pink. The final SP is depicted in magenta.

The SP was identified by cutting out the part of the sacrum between two sagittal planes originated in the PSISs. If the most anterior point of this part is located on the intersection of one of the planes, the point is discarded and the cutting plane is moved stepwise in medial direction. The procedure is repeated until the most anterior point is not located on the intersection and thus defines the SP (Fig. 6).

#### Curvature method

A curvature-based approach was implemented in order to compare the detection rates and accuracies of both methods. It is based on two papers by Subburaj *et al*., the first one on pelvic landmark identification^18^ and the second one on landmark detection of the knee that describes the method in more detail^23^. Based on its curvature, the surface of the pelvis is partitioned into different types of regions: peaks, ridges, pits and ravines. Thereafter, the landmarks are identified in an iterative process by labeling the regions using a predefined spatial relationship matrix. The spatial relationship matrix defines the type of region of a landmark and constrains the relative positions of the landmarks to each other^23^.

The initialization of the spatial relationship matrix depends on the reference system of the pelvis. Subburaj *et al*. indicate that this reference system is the CT coordinate system. However, Subburaj *et al*. did not take into account that the anatomical planes of the patient can highly deviate from the CT coordinate system and that reference systems of medical imaging systems are not standardized. In contrast to the ITP method, Subburaj’s curvature method can therefore only be applied if the reference system is known. In order to ensure a unified reference system of the pelvis for the initialization of the spatial relationship matrix, independent from the medical imaging system and the patients’ alignment, the APP coordinate system of the ITP method was used. Subburaj’s published spatial relationship matrix includes the ASISs, anterior inferior iliac spines (AIISs), PSISs, PTs, iliac pubic symphyses (IPYs), iliac ischial tuberosities (IITs), and iliac tubercles (ITs)^18^. Additional landmarks can be added to the matrix. However, each landmark of the spatial relationship matrix represents a necessary condition that must be fulfilled. In order to ensure general validity for varying subject morphologies, two additional checks were added to the spatial relationship matrix that are not described in Subburaj’s papers. Two different landmarks whose spatial relationship is close to a plane parallel to the origin planes of the used coordinate system can have a predefined positive or negative offset in the direction of the plane’s normal. For two different landmarks, whose spatial relationship in one dimension cannot be defined at all, the spatial relationship matrix considers an infinite positive and negative offset.

#### Manual method

Five medical experts identified the ASISs, AIISs, PTs, PSISs, PIISs, ISs, SP and the center of the sacral plateau on the pelvis surfaces using a graphical user interface implemented in MATLAB. Each observer processed all 20 pelvises. The identification was repeated four times by each observer. Each observer was urged to process a maximum of 10 different pelvises per day. The pelvises were presented to the observer in the CT coordinate system. The PS was calculated from the PTs as described in the chapter “ITP method”.

### Evaluation

For the manual method, intraclass correlation coefficients were used to examine the inter-observer reliability (two-way random effects, single measures, absolute agreement) and the intra-observer reliability (two-way mixed effects, single measures, absolute agreement). For five raters and an estimated intraclass correlation coefficient of 0.9, the number of subjects had to be larger than 10 ensuring a desired 95% confidence interval (α = 0.05) with a width of 0.2 mm^24^. The intraclass correlation coefficients were calculated for the CT coordinate system.

For the automatic methods, the detection rate is computed for each landmark or a pair of bilateral landmarks. The detection rate is defined as ratio of the number of detections of a landmark to the total number of this landmark for all subjects.

For the comparison of the manual method with the automatic methods and for the differentiation of the deviations in the anatomical directions within a unified coordinate system, the manual landmarks were transformed into the APP coordinate system calculated by the ITP method. The distributions of the manual landmarks were tested for normality using the Lilliefors test. For 29% of the variables the test was rejected and therefore non-parametric statistics were used hereafter. The median of all observers and all trials was calculated for each landmark and the nearest point on the surface of the pelvis to the median point was taken as the reference landmark. The reference landmarks were subtracted from the manual landmarks to investigate the intra- and inter-observer errors as well as from the automatic landmarks to evaluate the positional deviations between the manual method and the automatic methods. In order to determine the deviations in the orientation of the derived coordinate systems, Euler angles were computed using the rotation matrix to transform the coordinate system of the reference landmarks into the coordinate system of the investigated method. The construction of the APP and the SISP coordinate system is described in the chapter “ITP method”. The angles describe the deviations for the pelvic tilt (x-axis), the pelvic bend (y-axis) and the pelvic rotation (z-axis). Wilcoxon rank-sum test (α = 0.05) was used to identify significant differences between the methods.

## Results

Based on the lower boundary values of the 95% confidence interval of the intraclass correlation coefficients the inter-observer reliability (min. 0.906) as well as the intra-observer reliability (min. 0.903) of the manual method can be considered as excellent.

**Figure 7:**
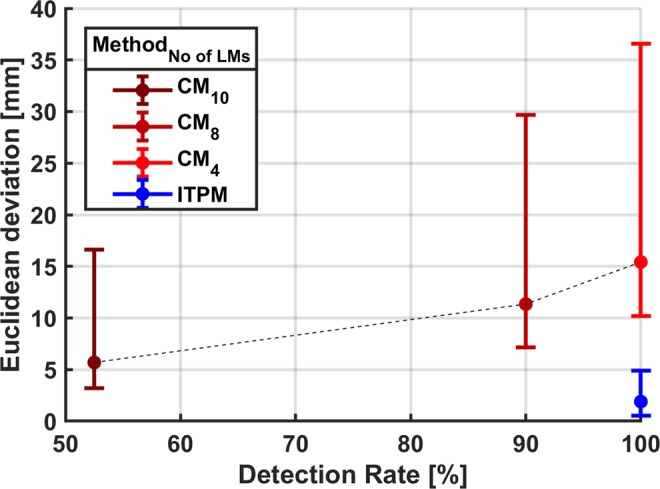
Relationship between the detection rate and the Euclidean deviation (Median, interquartile range) for the PSISs. The results of the curvature method (CM) for a varying number of landmarks (No of LMs) in the spatial relationship matrix are depicted in shades of red. The result for the ITP method (ITPM) is depicted in blue. CM_4_ detects the PS, ASISs, PSISs and ISs. In addition to the landmarks of CM_4_, CM_8_ detects the AIISs, IPYs, IITs and ITs. In addition to the landmarks of CM_8_, CM_10_ detects the PIISs and ISs. ITPM detects the PS, ASISs, PSISs, ISs and SP.

While the ITP method was able to detect all landmarks of all 20 pelvises, the detection rate of the curvature method showed to be dependent on the number of landmarks in the spatial relationship matrix. Figure 7 illustrates the relationship between the detection rate and the Euclidean deviation of the PSISs for a varying number of landmarks in the spatial relationship matrix of the curvature method. Landmarks that only can be detected by the curvature method were removed from the spatial relationship matrix. Hence, the spatial relationship matrix with the smallest number of landmarks contained only landmarks that could be detected by the curvature method as well as the ITP method. The PSISs were chosen for the comparison since they were the only landmarks that were part of Subburaj’s originally published spatial relationship matrix and were not part of the ITP method-based APP coordinate system that was used for the initialization of the spatial relationship matrix. Figure 7 shows that a lesser number of landmarks improves the detection rate of the curvature method albeit increasing the deviations to the reference landmarks. Due to this reason and the dependency of the curvature method on the unified APP coordinate system of the ITP method for the initialization of the spatial relationship matrix, only the ITP method was compared to the manual method in the further evaluation.

The robustness of the ITP method against the initial alignment of the pelvis in space was evaluated by applying a random transformation (rotation and translation) to the pelvis in the CT coordinate system and calculating the APP coordinate system afterwards. The procedure was repeated 100 times for each subject. For all subjects the positional deviation of the landmarks defining the APP between the 100 randomized repetitions was below 10^−11^ mm.

The median deviations between the landmarks determined by the ITP method (ITPMDs) and the reference landmarks range from −0.5 mm to 0 mm in medial-lateral direction, from 0 mm to 2.1 mm in posterior-anterior direction and from −1 mm to 3 mm in inferior-superior direction for different landmarks. The median manual method’s deviations (MMDs) to the reference landmark are 0 mm in medial-lateral as well as in inferior-superior direction, and range from −0.3 mm to 0.3 mm in posterior-anterior direction. For the Euclidean distance, the ITPMDs range from 1.9 mm to 3.4 mm compared to the MMDs from 1.1 mm to 2.9 mm. The following deviations are reported as median (interquartile range, min.-max. range) in mm. For the Euclidean distance of the PS for example, the MMD of 1.1 (1.1, 7.6) mm is significantly smaller than the ITPMD of 3.3 (2.4, 6.9) mm, whereas for the Euclidean distance of the PSISs the MMD of 2.9 (3, 22.6) mm is significantly larger than the ITPMD of 1.9 (1.7, 6.3) mm (Table 3).

**Table 3:** Deviations of the landmarks. Manual method’s deviation (MMD) (n = 400), ITP method’s deviation (ITPMD) (n = 20), p values of the Wilcoxon rank-sum test (α = 0.05).

Landmark	Direction	MMD [mm]			ITPMD [mm]			p value
		Median	IQR (Q1 to Q3)	Range (min. to max.)	Median	IQR (Q1 to Q3)	Range (min. to max.)	
ASISs	Med.-Lat.	0	2.5 (−1.3 to 1.2)	12.1 (−5.7 to 6.4)	−0.3	3.0 (−1.4 to 1.5)	9.5 (−5.9 to 3.6)	0.95
	Pos.-Ant.	−0.3	1.0 (−0.9 to 0.1)	13.1 (−11.0 to 2.1)	0.6	0.6 (0.3 to 0.8)	2.5 (0.0 to 2.5)	<0.05
	Inf.-Sup.	0	3.5 (−1.5 to 2.0)	29.6 (−13.4 to 16.3)	3	3.4 (1.7 to 5.1)	13.2 (−1.3 to 11.9)	<0.05
	Euc. Dist.	2.7	2.6 (1.6 to 4.2)	17.3 (0.0 to 17.3)	3.4	2.4 (2.8 to 5.3)	12.4 (0.0 to 12.4)	<0.05
PS	Med.-Lat.	0	0.4 (−0.2 to 0.2)	5.3 (−2.8 to 2.5)	0	0.7 (−0.4 to 0.3)	5.0 (−2.2 to 2.8)	0.71
	Pos.-Ant.	0	0.8 (−0.5 to 0.3)	11.1 (−7.4 to 3.7)	2.1	1.8 (1.1 to 2.9)	6.2 (0.6 to 6.8)	<0.05
	Inf.-Sup.	0	1.7 (−0.8 to 0.9)	9.5 (−4.2 to 5.4)	1.4	2.8 (0.7 to 3.5)	9.4 (−2.6 to 6.8)	<0.05
	Euc. Dist.	1.1	1.1 (0.7 to 1.8)	7.6 (0.0 to 7.7)	3.3	2.4 (2.5 to 4.8)	6.9 (1.4 to 8.3)	<0.05
PSISs	Med.-Lat.	0	2.9 (−1.6 to 1.3)	22.6 (−13.1 to 9.6)	−0.5	1.8 (−1.3 to 0.4)	5.3 (−3.2 to 2.0)	0.16
	Pos.-Ant.	0.3	0.8 (0.0 to 0.8)	8.2 (−1.0 to 7.2)	0	0.3 (−0.2 to 0.1)	1.7 (−0.8 to 0.9)	<0.05
	Inf.-Sup.	0	3.7 (−2.1 to 1.6)	29.1 (−20.4 to 8.7)	1.2	2.2 (0.1 to 2.3)	8.3 (−2.1 to 6.2)	<0.05
	Euc. Dist.	2.9	3.0 (1.8 to 4.7)	22.6 (0.0 to 22.6)	1.9	1.7 (1.3 to 3.0)	6.3 (0.0 to 6.3)	<0.05
ISs	Med.-Lat.	0	1.0 (−0.5 to 0.5)	8.1 (−4.0 to 4.1)	0	1.7 (−0.9 to 0.8)	6.0 (−2.2 to 3.8)	0.75
	Pos.-Ant.	0.1	0.8 (−0.1 to 0.6)	6.8 (−1.4 to 5.5)	0.9	0.7 (0.5 to 1.2)	2.2 (−0.2 to 2.0)	<0.05
	Inf.-Sup.	0	1.9 (−1.0 to 1.0)	16.2 (−8.8 to 7.3)	−1	1.9 (−2.4 to −0.5)	6.0 (−5.2 to 0.8)	<0.05
	Euc. Dist.	1.4	1.4 (0.9 to 2.3)	10.2 (0.0 to 10.2)	1.9	1.3 (1.5 to 2.7)	5.2 (0.7 to 5.9)	<0.05
SPs	Med.-Lat.	0	1.7 (−0.9 to 0.8)	10.3 (−4.8 to 5.5)	−0.3	3.4 (−2.4 to 1.0)	9.5 (−5.0 to 4.6)	0.22
	Pos.-Ant.	−0.1	0.3 (−0.3 to 0.0)	2.3 (−1.6 to 0.7)	0	0.3 (−0.1 to 0.2)	1.0 (−0.3 to 0.7)	<0.05
	Inf.-Sup.	0	1.1 (−0.5 to 0.6)	4.2 (−1.9 to 2.3)	0.7	1.2 (0.1 to 1.3)	3.2 (−0.6 to 2.7)	<0.05
	Euc. Dist.	1.1	0.8 (0.9 to 1.7)	5.5 (0.0 to 5.5)	1.9	2.4 (1.0 to 3.4)	5.1 (0.0 to 5.1)	<0.05

For the coordinate systems calculated from these landmarks (see chapter “ITP method”), the only significant difference between the MMDs and the ITPMDs can be observed for the pelvic tilt of the APP coordinate system. The range of the MMDs is considerably larger than the range of the ITPMDs (Table 4).

**Table 4:** Deviations of the coordinate systems. Manual method’s deviation (MMD) (n = 400), ITP method’s deviation (ITPMD) (n = 20), p values of the Wilcoxon rank-sum test (α = 0.05).

CS	Rotation	MMD [°]			ITPMD [°]			p value
		Median	IQR (Q1 to Q3)	Range (min. to max.)	Median	IQR (Q1 to Q3)	Range (min. to max.)	
APP	Pelvic Tilt	−0.1	0.9 (−0.6 to 0.3)	8.4 (−4.7 to 3.7)	−0.7	1.2 (−1.3 to −0.1)	4.0 (−3.6 to 0.3)	<0.05
	Pelvic Bend	0	0.8 (−0.5 to 0.4)	5.9 (−3.5 to 2.4)	−0.1	1.2 (−0.4 to 0.7)	2.7 (−0.9 to 1.8)	0.73
	Pelvic Rot.	0	0.3 (−0.1 to 0.2)	3.9 (−1.1 to 2.8)	0	0.1 (−0.1 to 0.1)	0.8 (−0.5 to 0.3)	0.83
SISP	Pelvic Tilt	−0.1	2.0 (−1.3 to 0.7)	10.8 (−7.3 to 3.4)	−0.9	1.2 (−1.3 to −0.2)	4.7 (−3.6 to 1.0)	0.06
	Pelvic Bend	0	0.8 (−0.5 to 0.4)	6.0 (−3.6 to 2.4)	−0.1	1.2 (−0.4 to 0.7)	2.7 (−0.9 to 1.8)	0.73
	Pelvic Rot.	0	0.3 (−0.1 to 0.2)	3.4 (−1.0 to 2.4)	0	0.1 (−0.1 to 0.1)	0.8 (−0.5 to 0.3)	0.75

The corresponding box plots can be found as Supplementary Fig. S3.

## Discussion

While the evaluation of Subburaj’s curvature method identified a trade-off between detection rate and accuracy, the ITP method had a detection rate of 100% and proved to be more accurate than the curvature method. A spatial relationship matrix with a higher number of landmarks does not seem to cover the large inter-individual variability of the pelvic morphology reducing the detection rate. A spatial relationship matrix with a lower number of landmarks seems to be insufficiently constrained for an accurate identification of the landmarks. Additionally, the necessity of a unified reference system for the initialization of the spatial relation matrix could only be fulfilled by using the APP coordinate system of the ITP method. For knee landmarks, Subburaj *et al*. reported an error of the curvature method lower than the inter-observer error for three subjects^23^. This leads to the idea, that a curvature-based refinement of the landmarks of the ITP method could potentially reduce the ITPMDs compared to the MMDs.

For the ASISs, the PS, the ISs and the SP, the ITPMDs are significantly higher than the MMDs in posterior-anterior direction, inferior-superior direction and for the Euclidean distance. For the ASISs and the PS (that is constructed by the PTs), this results mainly from the fact that the contact points of the APP, defined by placing the anterior side of the pelvis on a table, are in many cases not the manually derived reference points of the ASISs or the PTs. These contact points are often located more superior and anterior for the ASISs and additionally more medial for the PTs. This also explains the significant higher ITPMD for the pelvic tilt of the APP coordinate system. The MMDs of the Euclidean distance of the ASISs and the PS are similar to the inter-observer errors reported by Seim *et al*.^15^. The same accounts for the difference between ITPMDs and MMDs of ISs and the SP, where the manually selected points are often not the most anterior, most posterior or most medial points of the landmarks identified by the ITP method. In contrast, for the PSISs, the ITPMD is significantly smaller than the MMD for the Euclidean distance. This also results from the difficult manual identification of the PSISs that is illustrated by the largest range of the MMD for the Euclidean distance. Thus, the ITPMDs and MMDs for the orientation of the SISP coordinate system recommended by the International Society of Biomechanics show no significant differences.

Besides the reproducibility, the lower number of outliers is an advantage of the ITP method over the manual method. Considering these advantages and depending on the context of use, the significant differences between ITPMDs and MMDs might be acceptable or neglectable. This has to be evaluated for each specific use case combining the ITP method and the manual method. For instance, the differences might be acceptable within a framework that includes a pre-registration based on landmarks derived from the ITP method and the manual method followed by a surface based refinement of the registration. The differences should be neglectable in cases where solely the ITP method is used, for instance the construction of statistical shape models or automated morphometric analyses of many subjects from different databases.

In this study, the methods were tested with fully reconstructed pelvises with closed outer surfaces of the hip bones and the sacrum. A full reconstruction of the sacrum might not be available in many cases. However, the ITP method was applied to 201 datasets in an ongoing study with a partially reconstructed sacrum. After thresholding, only the areas of the SP and the sacral plateau were manually reconstructed if necessary. The preliminary results show no impact of the partial reconstruction of the sacrum on the robustness of the ITP method. A partial reconstruction of the hip bones was not evaluated.

Large osteophytes, severe bone deformities, bone tumors, orthopedic surgeries like osteotomies, bone fractures with large displacements of the bone fragments or metal artifacts that were not removed during the reconstruction process of the surface model can cause misdetections of specific landmarks. Supplementary Text S4 contains three-dimensional figures of the only five subjects out of a database of 228 pelvises with an obvious misdetection of a single landmark whereas the remaining landmarks were properly identified. However, such severe changes in the pelvic morphology would very likely also affect the results of other methods. The five subjects have either large osteophytes, severe bone deformities or triple pelvic osteotomies. Two additional cases with triple pelvic osteotomies are shown that did not affect the ITP method.

In conclusion, this study presents a robust fully automatic method for identification of landmarks on surface models of the pelvis. The method detects the landmarks to construct the two most established bone coordinate systems of the pelvis based on the APP and the SISP. Whether the minor but significant differences between the ITP method and the manual method for the landmarks and the APP coordinate system are acceptable, has to be evaluated for each specific use case. If these differences can be reduced by an additional curvature-based refinement is part of our ongoing work.

## Supplementary information

Supplementary Table S1

Supplementary Table S2

Supplementary Fig. S3

Supplementary Text S4

## Data Availability

All data and code to reproduce the results of this study are openly accessible. A list of the subjects is provided in the Supplementary Table S2. The segmentations and reconstructions are available at http://www.smir.ch. The surface models^25^, the manually selected landmarks^25^ and the MATLAB code^26,27^ are published online. Future updates can be found at github.com/RWTHmediTEC.
