# Supplementary material for: A robust method for automatic identification of landmarks on surface models of the pelvis
Source: Sci Rep. 2019 Sep 16;9:13322. doi: 10.1038/s41598-019-49573-4 (PMC6746744; doi:10.1038/s41598-019-49573-4)
Supplement: Supplementary file 1 — Supplementary Table S1 [file 41598_2019_49573_MOESM1_ESM.pdf]

2019

A robust method for automatic identification of landmarks on surface models of the pelvis

SUPPLEMENTARY TABLE S1 - LIST OF ABBREVIATIONS

Maximilian C. M. Fischer, Felix Krooß, Juliana Habor, Klaus Radermacher
Chair of Medical Engineering, Helmholtz-Institute for Biomedical Engineering, RWTH Aachen University, Germany

Supplementary Table S1. List of abbreviations. In the paper, an "s" at the end of the abbreviation indicates the plural form.

**Landmarks**	
AIIS	anterior inferior iliac spine
ASIS	anterior superior iliac spine
IIT	iliac ischial tuberosity
IPY	iliac pubic symphysis
IS	ischial spine
IT	iliac tubercle
PIIS	posterior inferior iliac spine
PS	pubic symphysis
PSIS	posterior superior iliac spine
PT	pubic tubercle
SP	sacral promontory
**Coordinate systems**	
APP	anterior pelvic plane
CS	coordinate system
SISP	superior inferior spine plane
TPCS	temporary pelvic coordinate system
**Landmark identification methods**	
CM	curvature method
ITPM	iterative tangential plane method
MM	manual method
**Error metrics**	
ITPMD	median deviation between the landmarks determined by the MM and the reference landmarks
MMD	median deviation between the landmarks determined by the ITPM and the reference landmarks
**Others**	
CT	computed tomography
No of LMs	number of landmarks
SICAS	Swiss Institute for Computer Assisted Surgery
SSM	statistical shape model
