# Supplementary material for: A robust method for automatic identification of landmarks on surface models of the pelvis
Source: Sci Rep. 2019 Sep 16;9:13322. doi: 10.1038/s41598-019-49573-4 (PMC6746744; doi:10.1038/s41598-019-49573-4)
Supplement: Supplementary file 2 — Supplementary Table S2 [file 41598_2019_49573_MOESM2_ESM.pdf]

2019

A robust method for automatic identification of landmarks on surface models of the pelvis

SUPPLEMENTARY TABLE S2 – INFORMATION ON THE CADAVERIC SUBJECTS

Maximilian C. M. Fischer, Felix Krooß, Juliana Habor, Klaus Radermacher

Chair of Medical Engineering, Helmholtz-Institute for Biomedical Engineering, RWTH Aachen University, Germany

Supplementary Table S2. Used cadaveric subjects from the open source virtual skeleton database (VSDFullBody) hosted at www.smir.ch.

**Number**	z001	z009	z013	z019	z023	z024	z027	z035	z036	z042	z046	z049	z050	z055	z056	z057	z061	z062	z064	z066
**Age**	76	25	41	58	47	57	37	30	62	61	38	34	84	73	26	75	39	43	69	48
**Gender**	M	M	F	M	F	F	F	F	M	F	M	M	M	M	M	M	F	M	M	M
