# Supplementary material for: A robust method for automatic identification of landmarks on surface models of the pelvis
Source: Sci Rep. 2019 Sep 16;9:13322. doi: 10.1038/s41598-019-49573-4 (PMC6746744; doi:10.1038/s41598-019-49573-4)
Supplement: Supplementary file 3 — Supplementary Fig. S3 [file 41598_2019_49573_MOESM3_ESM.pdf]

2019

A robust method for automatic identification of landmarks on surface models of the pelvis

SUPPLEMENTARY FIGURES S3 – DEVIATIONS OF LANDMARKS AND COORDINATE SYSTEMS

Maximilian C. M. Fischer, Felix Krooß, Juliana Habor, Klaus Radermacher
Chair of Medical Engineering, Helmholtz-Institute for Biomedical Engineering, RWTH Aachen University, Germany

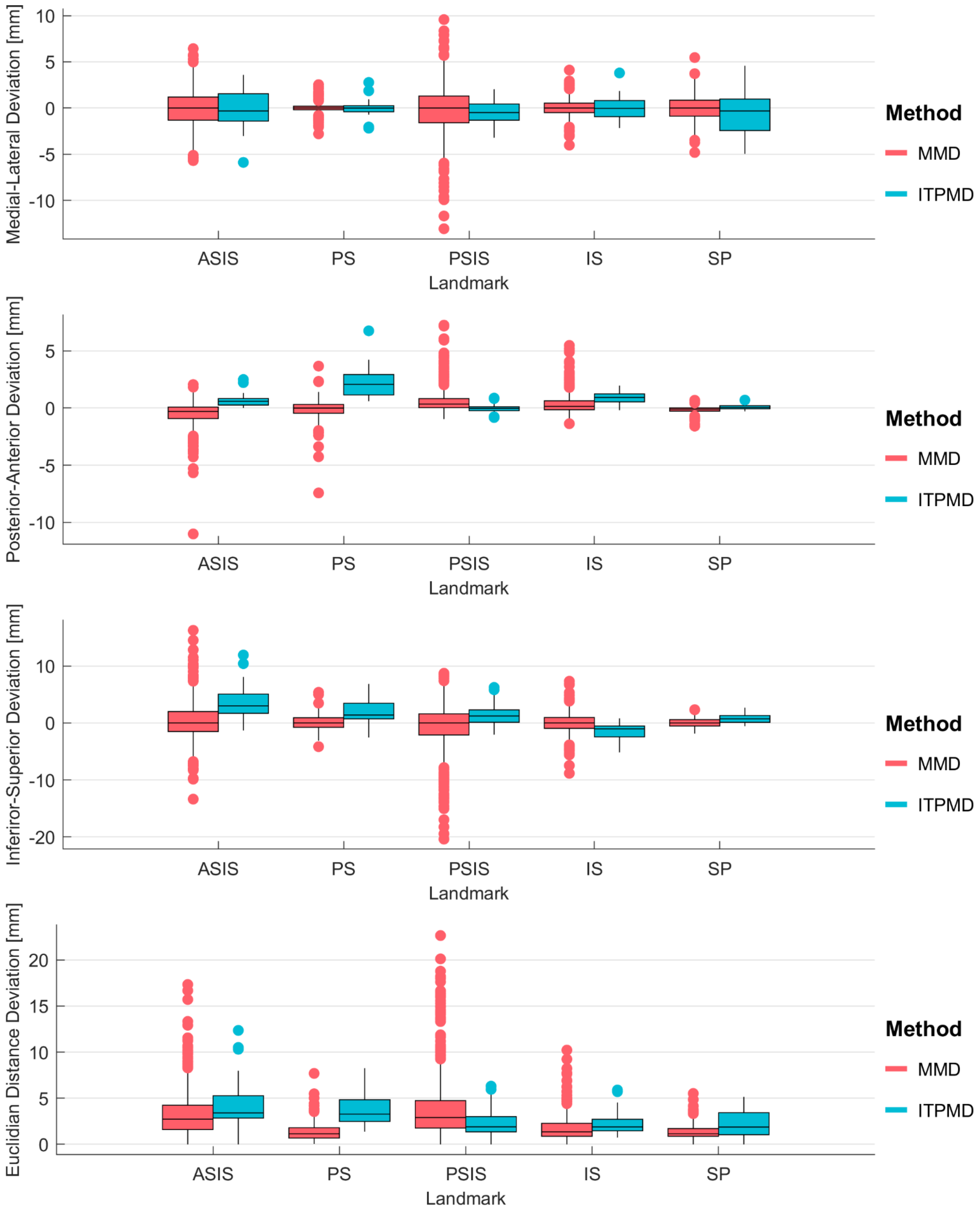

Supplementary Figure S3.1. Deviations of the landmarks. Manual method's deviation (MMD) (n = 400), ITP method's deviation (ITPMD) (n = 20).

Box plot of Table 3 in the paper.

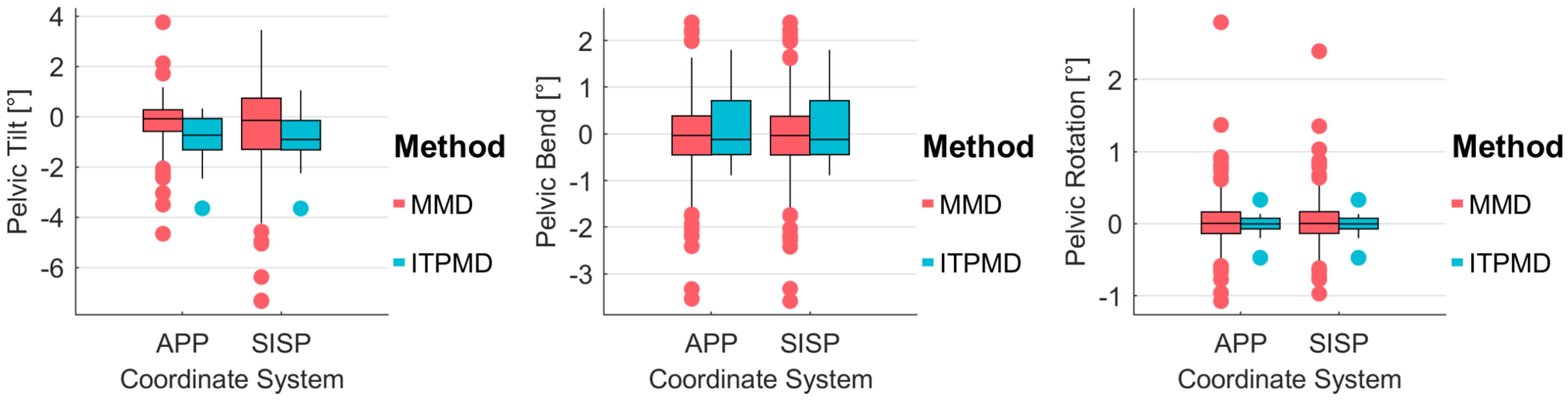

Supplementary Figure S3.2. Deviations of the coordinate systems. Manual method's deviation (MMD) (n = 400), ITP method's deviation (ITPMD) (n = 20). Box plot of Table 4 in the paper.
