# Supplementary material for: A robust method for automatic identification of landmarks on surface models of the pelvis
Source: Sci Rep. 2019 Sep 16;9:13322. doi: 10.1038/s41598-019-49573-4 (PMC6746744; doi:10.1038/s41598-019-49573-4)
Supplement: Supplementary file 4 — Supplementary Text S4 [file 41598_2019_49573_MOESM4_ESM.pdf]

A robust method for automatic identification of landmarks on surface models of the pelvis

Supplement Text S1 - Cases with large osteophytes, severe deformities or triple pelvic osteotomies

Maximilian C. M. Fischer, Felix Krooß, Juliana Habor, Klaus Radermacher

2019

Contents

1 Cases with large osteophytes, severe deformities or triple pelvic osteotomies 3
1.1 ASIS misdetection 4
1.2 IS misdetection 6
1.3 SP misdetection 7
1.4 Triple pelvic osteotomy examples 9

List of Figures 11

Chapter 1

Cases with large osteophytes, severe deformities or triple pelvic osteotomies

This document contains the only five subjects out of a database of 228 pelvises with obvious misdetections by the ITP method. Additionally, it contains two examples of subjects with triple pelvic osteotomies that did not affect the automatic landmark identification by the ITP method. All cases were visually inspected by one experienced observer after applying the ITP method.

**Landmarks**	**Color**
ASISs	Yellow
PS	Blue
PSISs	Red
ISs	Green
SP	Magenta

Table 1.1: Colors of the landmarks in this document

Warning: Loading of the next pages might take some time!

1.1 ASIS misdetection

The only two out of 228 subjects with an obvious misdetection of the ASIS.

Figure 1.1: Misdetection of the left ASIS due to very large anterior acetabular osteophytes.

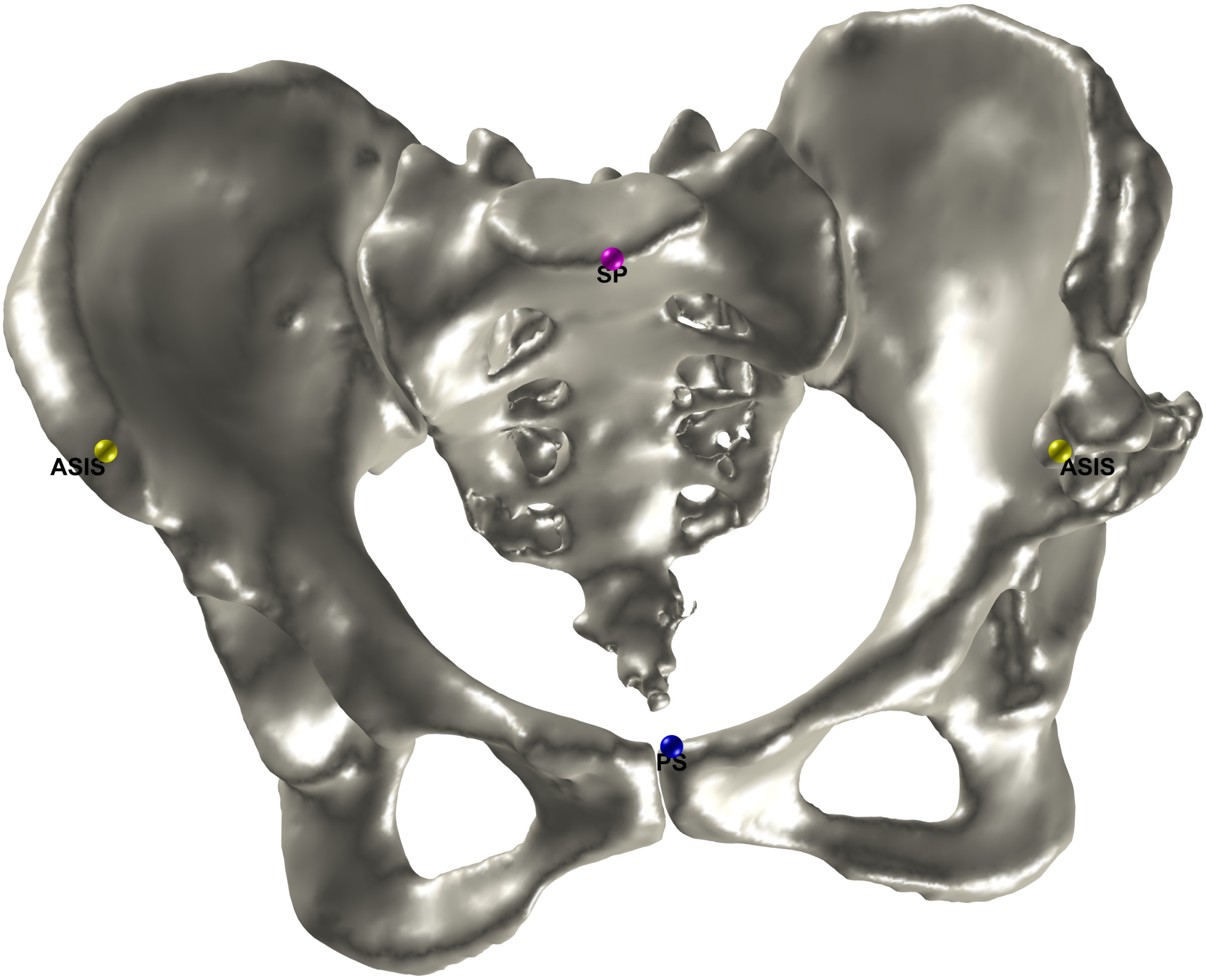

Figure 1.2: Misdetection of the right ASIS due to a triple pelvic osteotomy.

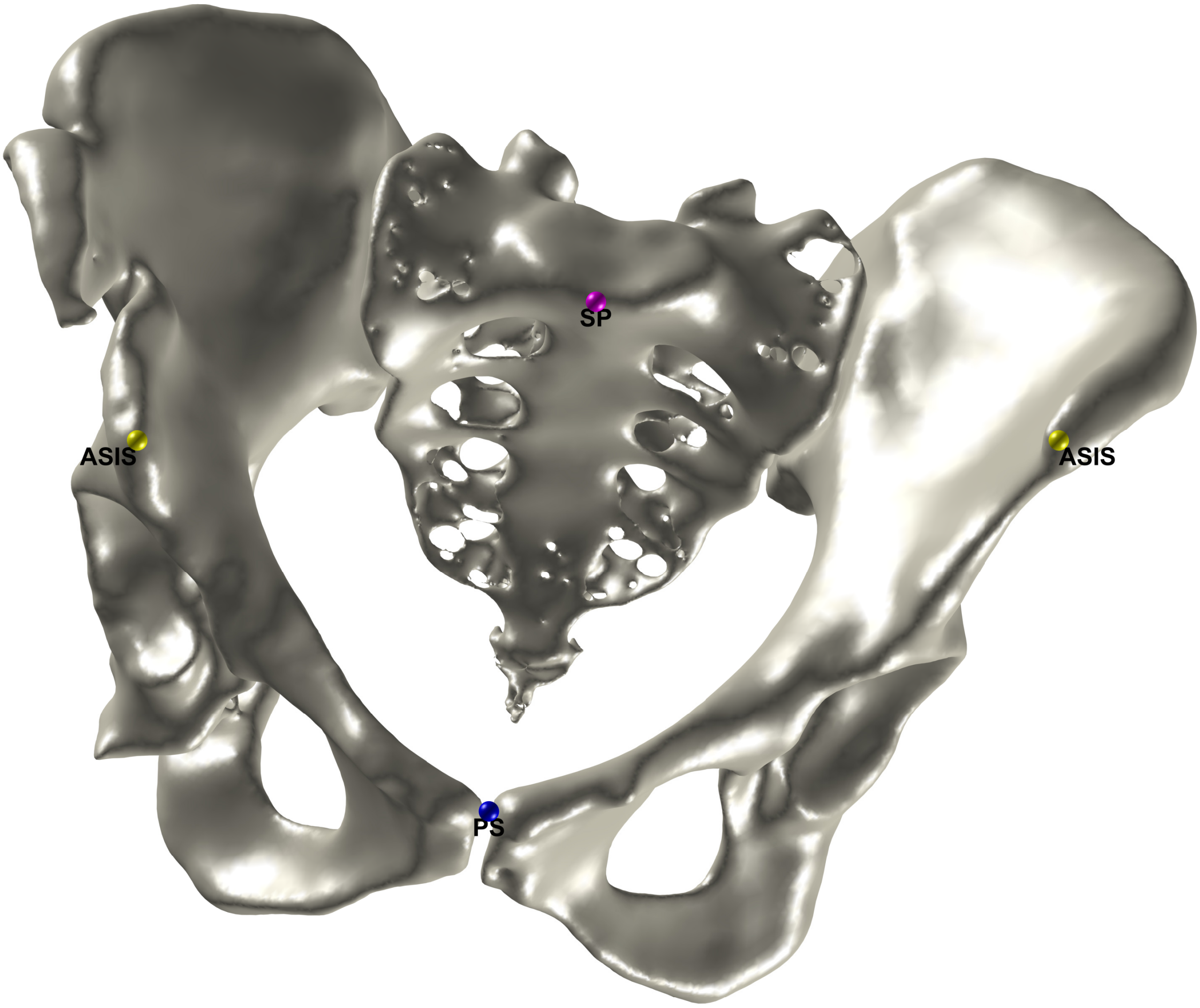

1.2 IS misdetection

The only one out of 228 subjects with an obvious misdetection of the IS.

Figure 1.3: Misdetection of the right IS due to a deformed sacrum. Pelvis with sacroiliac ankylosis.

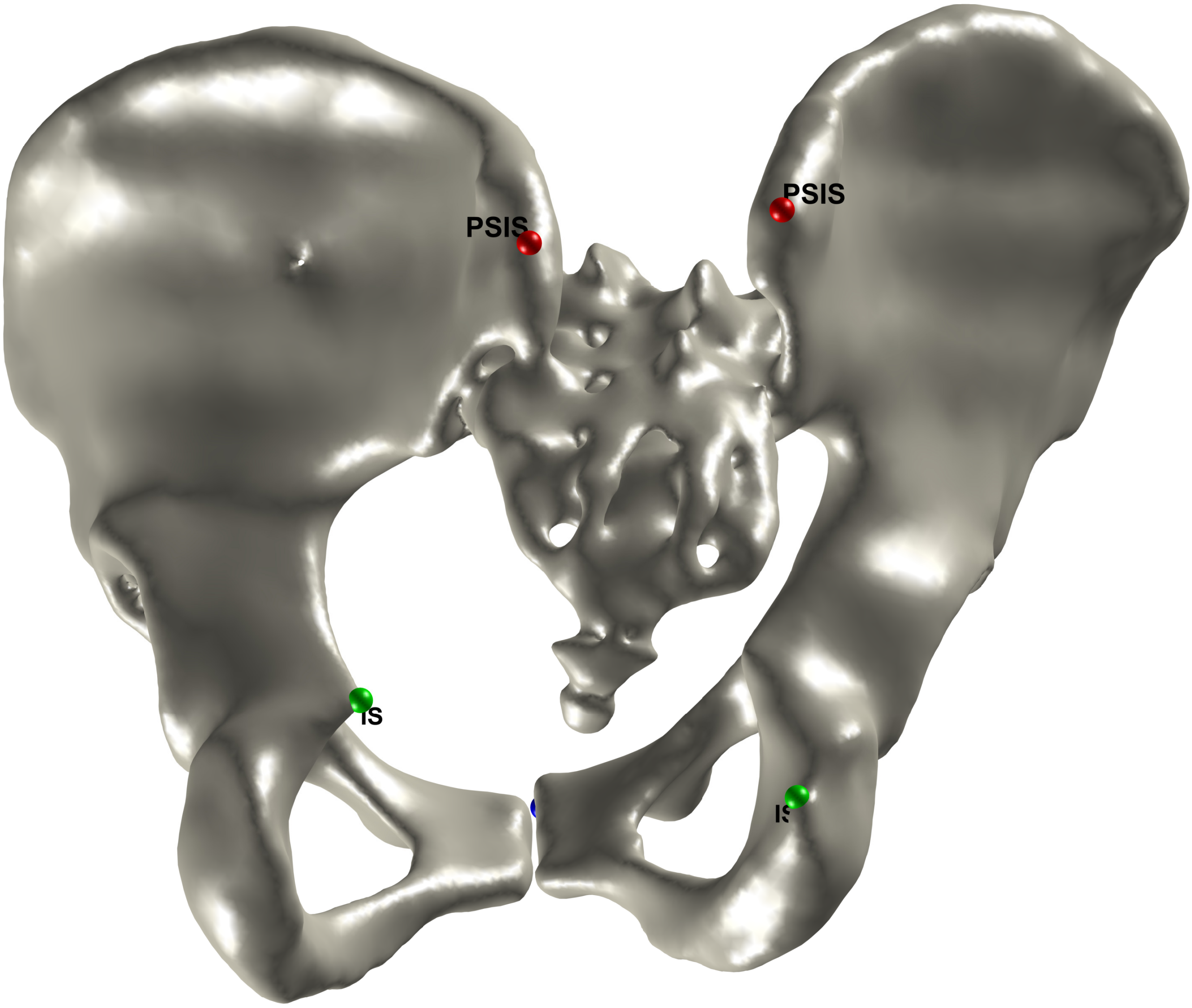

1.3 SP misdetection

The only two out of 228 subjects with an obvious misdetection of the SP.

Figure 1.4: Misdetection of the SP due to osteophytes at sacral plateau.

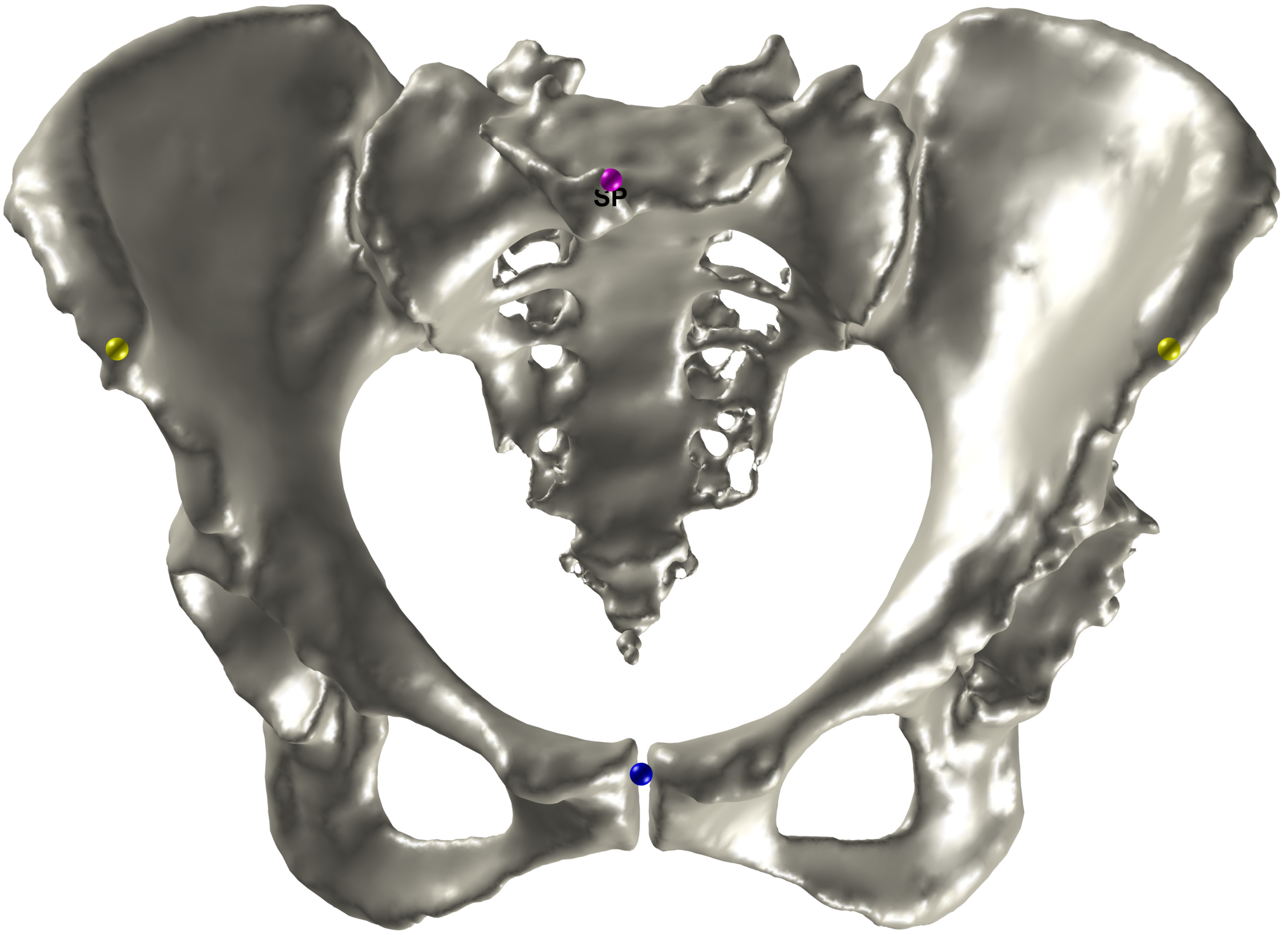

Figure 1.5: Misdetection of the SP due to osteophytes at sacral plateau. Pelvis with sacroiliac ankylosis.

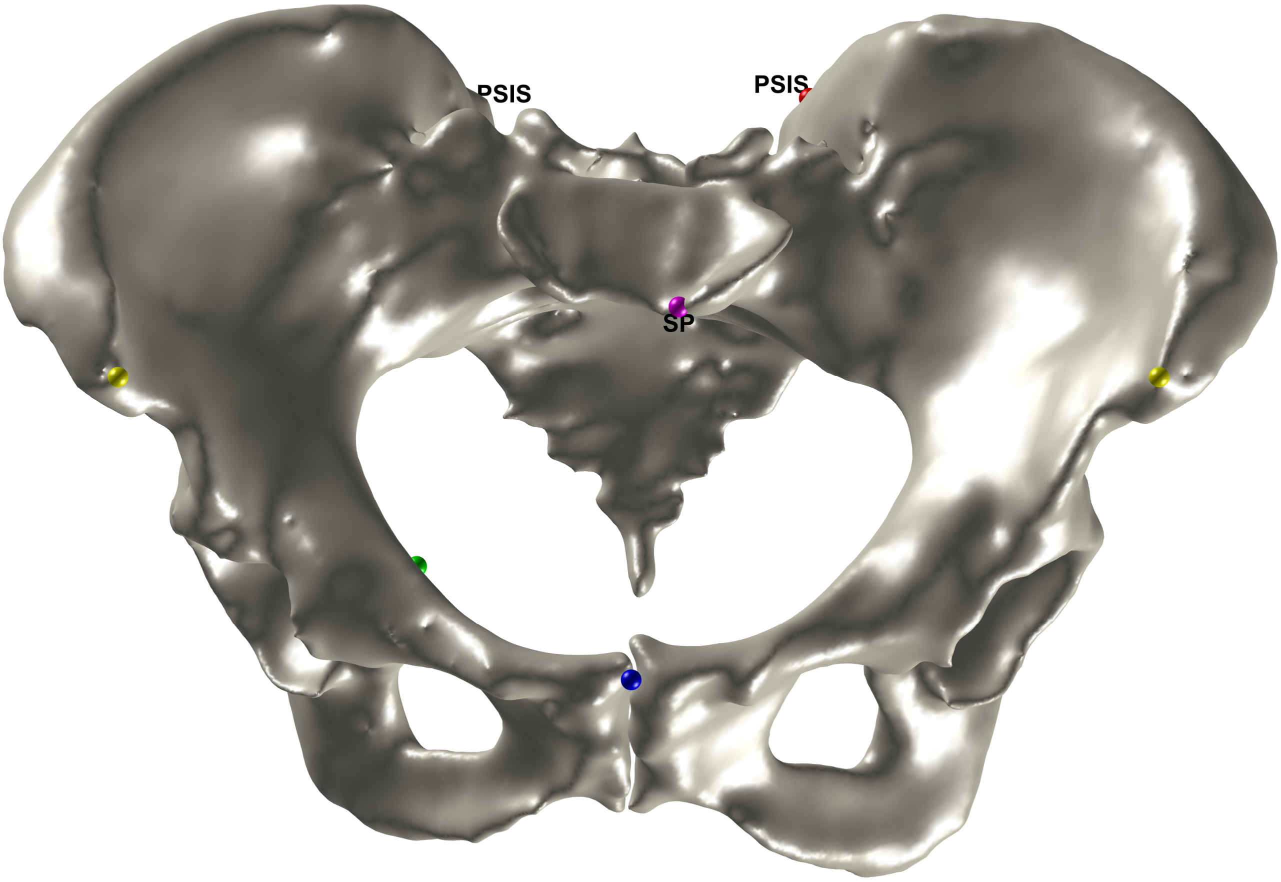

1.4 Triple pelvic osteotomy examples

Two examples of subjects with triple pelvic osteotomies that did not affect the ITP method.

Figure 1.6: Subject with an triple pelvic osteotomy on both sides.

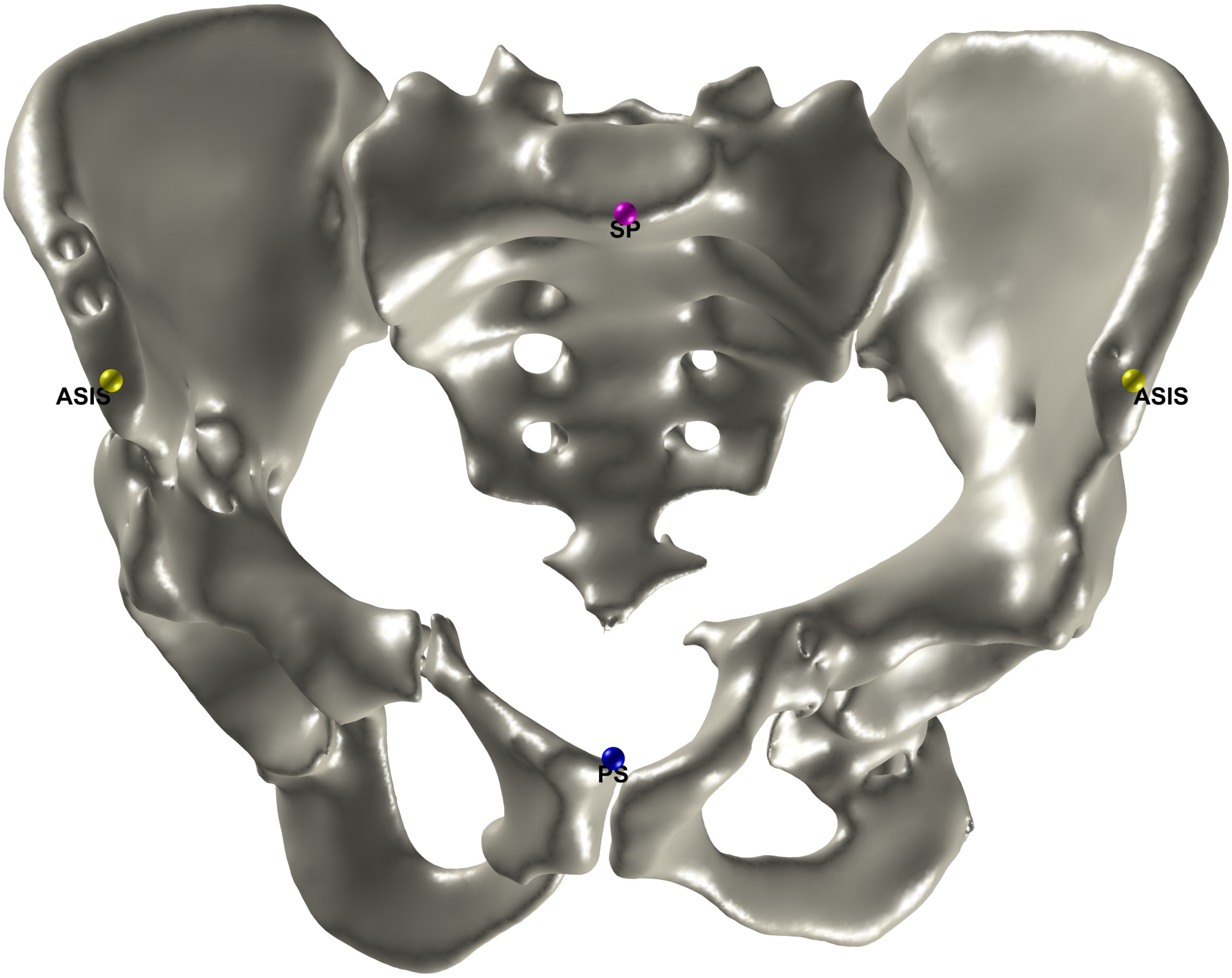

Figure 1.7: Subject with an triple pelvic osteotomy on the left side.

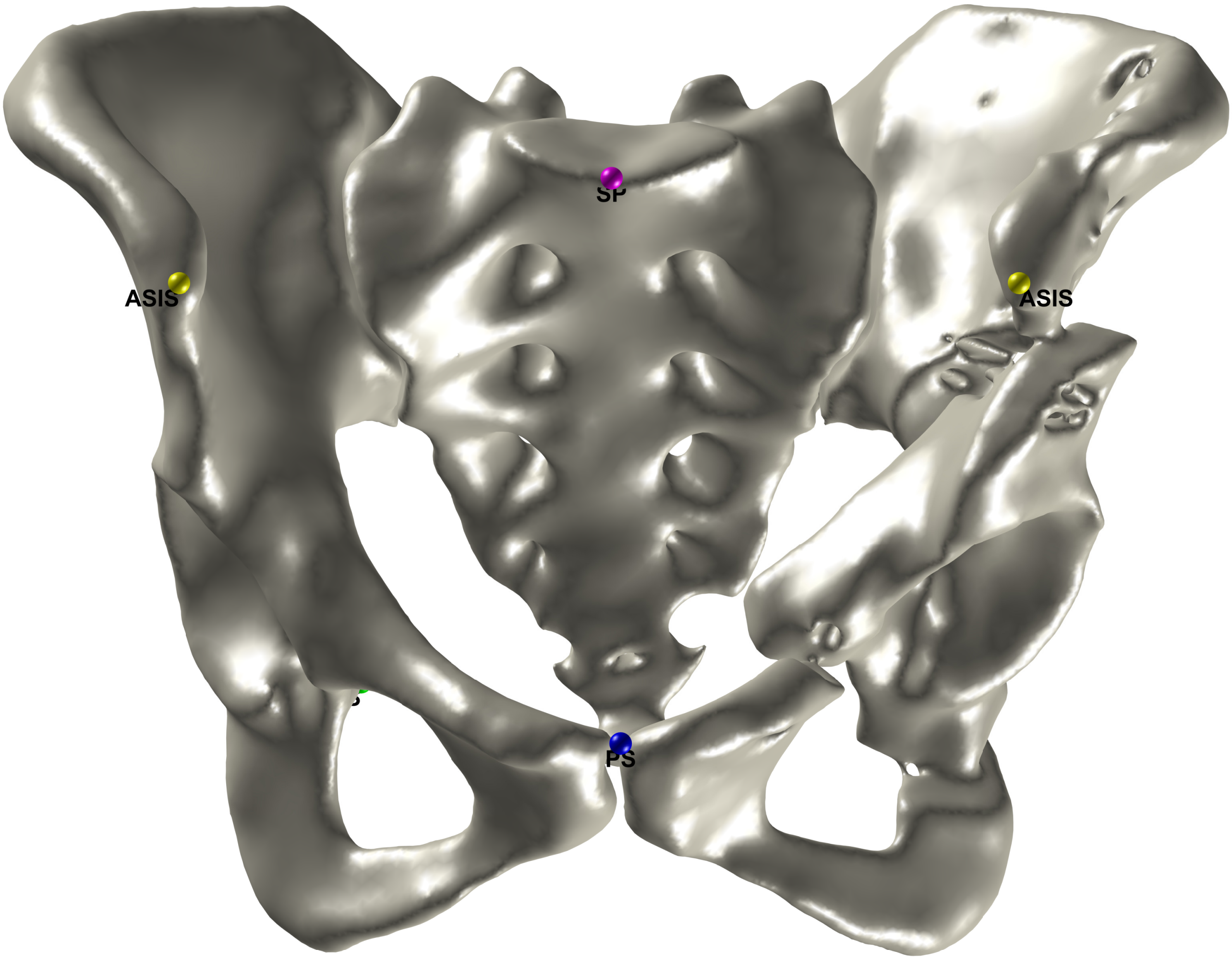

List of Figures

1.1 Misdetection of the left ASIS due to very large anterior acetabular osteophytes. 4
1.2 Misdetection of the right ASIS due to a triple pelvic osteotomy. 5
1.3 Misdetection of the right IS due to a deformed sacrum. Pelvis with sacroiliac ankylosis. 6
1.4 Misdetection of the SP due to osteophytes at sacral plateau. 7
1.5 Misdetection of the SP due to osteophytes at sacral plateau. Pelvis with sacroiliac ankylosis. . . 8
1.6 Subject with an triple pelvic osteotomy on both sides. 9
1.7 Subject with an triple pelvic osteotomy on the left side. 10
